# Structural basis for the different states of the spike protein of SARS-CoV-2 in complex with ACE2

**DOI:** 10.1038/s41422-021-00490-0

**Published:** 2021-03-18

**Authors:** Renhong Yan, Yuanyuan Zhang, Yaning Li, Fangfei Ye, Yingying Guo, Lu Xia, Xinyue Zhong, Ximin Chi, Qiang Zhou

**Affiliations:** 1 Center for Infectious Disease Research, Westlake Laboratory of Life Sciences and Biomedicine, Key Laboratory of Structural Biology of Zhejiang Province, School of Life Sciences, Westlake University, Hangzhou, Zhejiang 310024 China; 2 Institute of Biology, Westlake Institute for Advanced Study, Hangzhou, Zhejiang 310024 China; 3 Beijing Advanced Innovation Center for Structural Biology, Tsinghua-Peking Joint Center for Life Sciences, School of Life Sciences, Tsinghua University, Beijing, 100084 China

**Keywords:** Cryoelectron microscopy, Mechanisms of disease

Dear Editor,

Severe acute respiratory syndrome coronavirus 2 (SARS-CoV-2) has become a severe threat to global health.^1^ The spike (S) protein on the surface of SARS-CoV-2 exploits angiotensin-converting enzyme 2 (ACE2) as cellular receptor and mediates the fusion of the viral and the host cell membrane during infection.^1,2^ The activation of the S protein requires the receptor-binding domain (RBD) binding to the peptidase domain (PD) of ACE2 and the cleavage by host proteases into the N-terminal S1 subunit and the C-terminal S2 subunit.^2–4^ An additional furin cleavage site “RRAR” present in the S1/S2 cleavage region of SARS-CoV-2, which is absent in SARS-CoV that shares about 80% sequence identity with SARS-CoV-2 and caused an epidemic in 2002–2003, might help accelerate the activation of the S protein. S1 consists of the N-terminal domain (NTD), the RBD, subdomain 1 and 2 (SD1 and SD2). The RBD adopts two distinct conformations: “up” and “down”. Only the “up” RBD exposes the receptor-binding site. S2, containing the hydrophobic fusion peptide, is responsible for the viral and cell membrane fusion. The molecular mechanism underlying the activation of the S protein of SARS-CoV-2 represents a critical clue for therapeutic development. Here we report the cryo-electron microscopy (cryo-EM) structures of trimeric extracellular domain of the S protein (hereinafter referred to as S) at multiple states and conformations, including the all RBD-locked, activated, S1/S2 trypsin-cleaved, PD of ACE2-bound, and the full-length ACE2-bound states. PD binds to the trimeric S protein with consistent interface, inducing conformational change at adjacent region. The trypsin-digested S protein tends to accommodate more PD molecules. Furthermore, the structure of the S–ACE2–B^0^AT1 ternary complex indicates that one ACE2 dimer binds two trimeric S proteins simultaneously. These results provide clues for the mechanism of the activation of the S protein of SARS-CoV-2.

We first solved the structure of the S protein alone that was purified in different batches and exhibited two conformations: the locked conformation and the active conformation, with overall resolutions of 2.7 Å and 3.3 Å, respectively. The locked conformation is similar to the previously reported structures,^5–8^ in which all RBD domains are in “down” position and compacted tightly, whereas in the active conformation, one of the RBD domains is in “up” position, which resembles the earliest reported structures^9,10^ (Fig. 1a; Supplementary information, Figs. S1–S6 and Table S1). We did not observe the previously reported closed conformation,^9^ in which all RBD domains stay in “down” position but pack looser than that in the locked conformation. Transformation from the locked to the active state implies a tendency for S1 to become loose (Supplementary information, Video S1). The contact area between RBD and NTD of the S protein is reduced from the “down” RBD in locked conformation (locked-down) to the “down” RBD in active conformation (active-down) and to the “up” RBD in the active conformation (active-up) (Fig. 1b). The S protein undergoes a clockwise twist from the locked conformation to the active conformation when looked from the S protein to the viral membrane, which loosens the NTD and SD1 domain by increasing the distance between the individual domain and the central axis (Fig. 1c). For clarity, the clockwise direction and position are defined according to the same view.

**Fig. 1:**
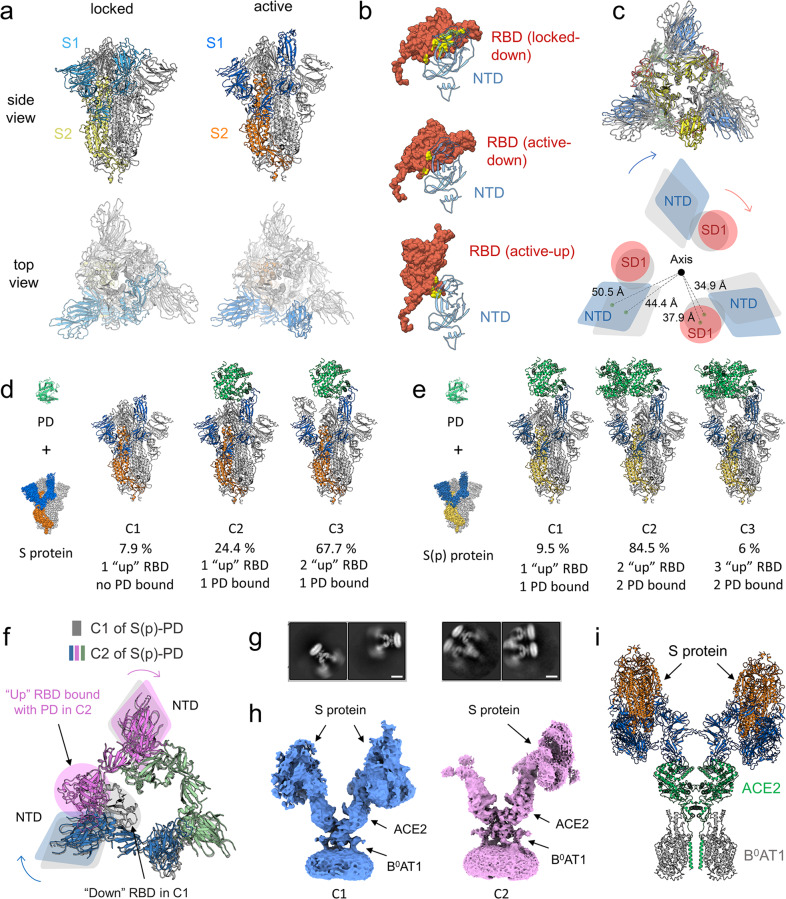
Characterization of the interactions of the S protein with ACE2. **a** Structures of the S protein. Left panel, locked conformation; right panel, active conformation with one RBD domain in the “up” position. **b** Decrease of contact area between RBD and NTD from the “down” RBD in the locked conformation (locked-down, upper panel), to the “down” RBD in the active conformation (active-down, middle panel), and to the “up” RBD in the active conformation (active-up, lower panel). **c** Structural comparison reveals a clockwise twist from the locked to the active conformation (upper panel), which loosens the contact between NTD and SD1 domain and increases the distance of these domains to the central axis. A schematic diagram is shown in the lower panel. Locked conformation is shown in gray. Active conformation is colored by domain, in which NTD is blue, RBD is yellow, SD1 is red and SD2 is green. **d**, **e** Different conformations of the S protein (**d**) and the S(p) protein (**e**) incubated with PD are shown, respectively. C1, C2 and C3 refer to conformation 1, 2 and 3, respectively. **f** Comparison between C1 and C2 of the S(p)–PD complex. Only RBD and NTD are shown for clarity. The binding of the second PD to RBD in C2 causes slight shift of NTDs both in the same protomer (violet) and in the anticlockwise protomer (blue). All protomers in C1 are colored in gray, whereas three protomers in C2 are colored in blue, violet and green, respectively. Circle and ellipse, RBD; diamond, NTD. **g** 2D class averages of the S–ACE2–B^0^AT1 ternary complex are shown. Scale bar, 10 nm. **h** Cryo-EM maps of conformation 1 (C1) and 2 (C2) of the S–ACE2–B^0^AT1 ternary complex. **i** Docking model of conformation 1 of the S–ACE2–B^0^AT1 ternary complex.

To investigate the activation mechanism of the S protein of SARS-CoV-2, we determined the structure of the S protein incubated with PD of ACE2 in three different conformations with overall resolutions of 3.0–3.5 Å (Fig. 1d; Supplementary information, Figs. S1–S5 and Table S1), which account for 7.9%, 24.4% and 67.7% of particles, respectively. In conformation 1, there is one “up” RBD, two “down” RBDs and no PD bound. In conformation 2, one RBD is in “up” conformation and bound by a PD molecule, whereas the other two RBDs are in “down” conformation. The conformation 2 resembles the previously reported S–PD complex.^7^ The conformation 3 is similar to conformation 2, except that the RBD clockwise near the PD-bound RBD is in “up” conformation and no PD molecule binds to it (Fig. 1d; Supplementary information, Video S2).

To investigate the effect of proteolytic processing on the activation of the S protein of SARS-CoV-2, we digested the purified S protein by trypsin, which is referred to as S(p) hereafter for clarity. We solved the structures of the S(p) protein in complex with PD of ACE2 (Fig. 1e; Supplementary information, Video S3) with overall resolutions of 2.9–3.6 Å, which show different PD binding results from the uncleaved S–PD complex. Three conformations of the S(p)–PD complex were observed. In conformation 1 that accounts for 9.5% particles, the S(p) trimer is bound by only one PD molecule with two RBDs in “down” state. In conformations 2 and 3 that respectively account for 84.5% and 6% particles, the S(p) trimer is bound by two PD molecules with the remaining RBD in “down” and “up” state, respectively. The binding of the second PD to the RBD of S(p) in conformation 2 induces shift of both the NTD in the same protomer and the anticlockwise NTD compared with conformation 1 (Fig. 1f; Supplementary information, Fig. S7). Moreover, we found that the released S1 of S(p) could also bind to PD (Supplementary information, Figs. S3b, S8), which is consistent with the previous study.^11^ Based on these discoveries, we propose that the cleavage at S1/S2 site might affect the architectural rigidity of the S protein, making RBD more accessible to ACE2.

Additionally, we solved the structures of the S(p), S(D614G), and the trypsin-digested S(D614G) (hereinafter referred to as S(p, D614G)) proteins with overall resolutions of 3.2 Å, 3.1 Å and 3.3 Å, respectively, which all exhibit one RBD “up” conformation and show no obvious difference (Supplementary information, Fig. S9a). The result of the S(p) protein is consistent with the previous study, which suggests that cleavage at the S1/S2 site facilitates the open conformation of RBD.^4^ The SARS-CoV-2 (D614G) mutant is associated with increased viral load in COVID-19 patients.^12^ To our surprise, S(p, D614G) shows more digested bands, which means that the D614G mutation might increase the flexibility of the S protein (Supplementary information, Fig. S9b). It is also reported that the D614G mutation can change the conformation of the S protein to accommodate more PDs of ACE2.^13^ Taken together, the D614G mutation might increase the flexibility of the S protein and make it easier to be digested by host protease, which might contribute to the increased infectivity.

Since PD of ACE2 exists as a monomer, whereas the full-length ACE2 presents as a dimer, a critical question is that how the full-length ACE2 binds to the S protein. In order to investigate the interactions of the S protein with ACE2 in a condition more closely resembling the physiological condition, we tried to solve the structure of the S protein in complex with the full-length ACE2. We prepared and purified the S–full-length ACE2–B^0^AT1 ternary complex, which showed comigrating bands in size exclusion chromatography (SEC) analysis (Supplementary information, Fig. S10a). 2D classification exhibited blurred density on the top of each PD of ACE2 dimer, which was attributed to the S protein, suggesting that one ACE2 dimer can be bound by two S proteins simultaneously (Fig. 1g, left panel). After 3D classification and refinement, we obtained two conformations for the S–ACE2–B^0^AT1 ternary complex with resolutions of 8.3 Å and 4.4 Å, respectively (Fig. 1h; Supplementary information, Figs. S10c–f, S11–S14). The right PD of the ACE2 dimer in both conformations is clearly bound by one S protein, whereas the left PD of the ACE2 dimer is bound by a much more blurred S protein and by a much smaller density in conformation 1 and 2, respectively. The local resolutions for ACE2, the left S protein and the right S protein in conformation 1 were improved to 8.4 Å, 10.4 Å and 6.7 Å, respectively (Supplementary information, Figs. S11, S13, S14a, c), which allowed a docking model for the S–ACE2–B^0^AT1 ternary complex (Fig. 1i). This finding confirmed our previous hypothesis that one dimeric ACE2 might bind to two trimeric S proteins simultaneously (Fig. 1i; Supplementary information, Fig. S14a, c).^3^ Besides, the local resolutions for ACE2 and the right S protein in conformation 2 were improved to 4.4 Å and 4.3 Å, respectively (Supplementary information, Figs. S12–S14). As we were unable to determine the left density bound to ACE2 in conformation 2, we did not generate a model for this conformation.

The two PD domains in a dimeric full-length ACE2 cannot bind to the same trimeric S protein because of the steric constriction, unless the RBD undergoes dramatic rotation as suggested by a previous study.^14^ It is not excluded that another ACE2 molecule can bind to the remaining RBD of the S protein from another direction. Interestingly, we found additional 2D averages, in which multiple ACE2 dimers form a complex with different RBDs of one S protein (Fig. 1g, right panel), consistent with a model proposed in the previous study.^14^ Additionally, the flexibility of the S protein on the top of ACE2 and the previously reported transition of ACE2 between the open and closed conformations might be important factors for the activation of the S protein during infection.^3^

## Supplementary information

Supplementary Information

Supplementary information, Video S1

Supplementary information, Video S2

Supplementary information, Video S3

## Data Availability

Atomic coordinates and cryo-EM density maps of conformation 1 of the S–ACE2–B^0^AT1 ternary complex (PDB: 7DWX, whole map: EMD-30888, map focused on ACE2: EMD-30967, map focused on the left S protein: EMD-30966, map focused on the right S protein: EMD-30965) and conformation 2 of the S–ACE2–B^0^AT1 ternary complex (whole map: EMD-30968, map focused on ACE2: EMD-30970, map focused on the right S protein: EMD-30969) have been deposited to the Protein Data Bank (http://www.rcsb.org) and the Electron Microscopy Data Bank (https://www.ebi.ac.uk/pdbe/emdb/), respectively. The other PDB and EMDB IDs can be found in Supplementary information, Table S1.
