# Supplementary material for: Structural basis for the different states of the spike protein of SARS-CoV-2 in complex with ACE2
Source: Cell Res. 2021 Mar 18;31(6):717–9. doi: 10.1038/s41422-021-00490-0 (PMC7972335; doi:10.1038/s41422-021-00490-0)
Supplement: Supplementary file 1 — Supplementary Information [file 41422_2021_490_MOESM1_ESM.pdf]

Materials and methods

Protein expression and purification

The extracellular domain (ECD) (1-1208 a.a) of S protein of SARS-CoV-2 (Genebank ID: QHD43416.1) was cloned into the pCAG vector (Invitrogen) with two proline substitutions at residues 986 and 987 and a C-terminal T4 fibritin trimerization motif followed by one Flag tag. This construct will hereafter be referred to as S(p). A "GSAS" mutation at residues 682 to 685 was introduced into S(p) to prevent the host furin protease digestion, which was referred to as S. A "D614G" mutation introduced into S(p) or S were referred to as S(p, D614G) or S(D614G) ,The cDNAs of full-length human B^0AT1 (accession number: NM_001003841) and ACE2 (accession number: NM_001371415) were subcloned into pCAG respectively. An N-terminal FLAG tag was fused to B^0AT1, and a Strep tag was fused after the N-terminal signal peptide of ACE2.

The peptidase domain (PD) (19-615 a.a) of human ACE2 was also cloned into the pCAG vector (Invitrogen) with an N-terminal signal peptide of secreted luciferase and a C-terminal Flag tag. The mutants were generated with a standard two-step PCR-based strategy. All the plasmids used to transfect cells were prepared by GoldHi EndoFree Plasmid Maxi Kit (CWBIO).

The purification processes of the S protein and ACE2-B^0AT1 complex have been described previously [1,2]. The purification protocol of S(D614G) was same as S. The S(p), S(p, D614G) and PD were purified as below: The recombinant protein was overexpressed using the HEK 293F mammalian cells (Invitrogen) at 37°C under 5% CO_2 in a Multitron-Pro shaker (Infors, 130 rpm). When the cell density reached 2.0 $\times 10^6$ cells/mL, the plasmid was transiently transfected into the cells. To transfect one liter of cell culture, about 1.5 mg of the plasmid was premixed with 3 mg of polyethylenimines (PEIs) (Polysciences) in 50 mL of fresh medium for 15 mins before adding to cell culture. Cells were removed by centrifugation at 4000×g for 15 mins after sixty hours transfection. The secreted proteins were purified by anti-FLAG M2 affinity resin (Sigma Aldrich). After loading two times, the anti-FLAG M2 resin was washed with the wash buffer containing 25 mM Tris (pH 8.0), 150 mM NaCl.

The protein was eluted with the wash buffer plus 0.2 mg/mL flag peptide. The eluent of PD was then concentrated and subject to size-exclusion chromatography (Superdex 200 Increase 10/300 GL, GE Healthcare) in buffer containing 25 mM Tris (pH 8.0), 150 mM NaCl.

The eluent of S(p) and S(p, D614G) were concentrated and digested by Trypsin TPCK treated (SCIEX) at a mass ratio of about 100:1 at 4℃ for two hours. Then the digestion was terminated by PMSF (Solarbio). The mixture was subject to size-exclusion chromatography (Superose 6 Increase 10/300 GL, GE Healthcare) in buffer containing 25 mM Tris (pH 8.0), 150 mM NaCl and the peak fractions were collected for EM analysis and generation of S(p) -PD complex.

The S(p) or S was incubated with PD at a molar ratio of about 1:3.6 for one hour. Then the mixture was subject to size-exclusion chromatography (Superose 6 Increase 10/300 GL, GE Healthcare) in buffer containing 25 mM Tris (pH 8.0), 150 mM NaCl.

The S protein was incubated with ACE2-B^0AT1 complex at a molar ratio of about 1.2:1 for two hours. And the mixture was also subject to size-exclusion chromatography (Superose 6 Increase 10/300 GL, GE Healthcare) in buffer containing 25 mM Tris (pH 8.0), 150 mM NaCl and 0.02% glyco diosgenin (GDN, Anatrace). The peak fractions were collected for EM analysis.

Cryo-EM sample preparation and data acquisition

S alone, S(p) alone, S(D614G) alone, S(p, D614G) alone, S-PD complex and S(p)-PD complex was concentrated to ~1.5 mg/mL and applied to the grids. Aliquots (3.3 μL) of the protein were placed on glow-discharged holey carbon grids (Quantifoil Au R1.2/1.3). The S protein and the ACE2-B^0AT1 complex was applied to Lacey Carbon Coated Grids (UC-A on lacey). The grids were blotted for 2.5 s or 3.0 s and flash-frozen in liquid ethane cooled by liquid nitrogen with Vitrobot (Mark IV, Thermo Fisher Scientific). The prepared grids were transferred to a Titan Krios operating at 300 kV equipped with Gatan K3 detector and GIF Quantum energy filter. Movie stacks were automatically collected using AutoEMation [3], with a slit width of 20 eV on the energy filter and a defocus range from -1.2 µm to -2.2 µm in super-resolution mode at a

nominal magnification of 81,000×. Each stack was exposed for 2.56 s with an exposure time of 0.08 s per frame, resulting in a total of 32 frames per stack. The total dose rate was approximately 50 $e^{-}/Å^{2}$ for each stack. The stacks were motion corrected with MotionCor2 [4] and binned 2-fold, resulting in a pixel size of 1.087 Å/pixel. Meanwhile, dose weighting was performed [5]. The defocus values were estimated with Gctf [6].

Data processing

Cryo-EM data was processed similarly for all S-related samples. Particles were automatically picked using Relion 3.0.6 [7-10] from manually selected micrographs. After 2D classification with Relion, good particles were selected and subject to one cycle of heterogeneous refinement without symmetry using cryoSPARC [11].The good particles were selected and subject to homogeneous refinement with C1 symmetry, resulting in the 3D reconstruction for the whole structures. The map quality of overall structure was improved by 3D refinement with Relion. Then, these particles were subject to 3D classification, 3D refinement and post-processing to catch the different conformations. For the S-ACE2-B^{0}AT1 complex, the methods for particle picking and 2D classification are same to that for the S protein, but the box size is 480 pixel. The good particles selected from 2D classification were subject to multiple heterogeneous refinement without symmetry using cryoSPARC, the last run of which resulted in two good classes. They were subject to non-uniform refinement (Legacy) without symmetry resulting in two conformations for the S-ACE2-B^{0}AT1 ternary complex. To further improve the map quality, the particles which contributed to the above two conformations, were re-extracted at the location of recognizable S protein and ACE2 and subjected to non-uniform refinement (Legacy) as the processing of the whole structure.

The resolution was estimated with the gold-standard Fourier sFhell correlation 0.143 criterion [12] with high-resolution noise substitution [13]. Refer to Supplemental information, Fig. S1, S2, S3, S4, S10, S11, S12, S13 and Table S1 for details of data collection and processing.

Model building and structure refinement

Model building of the S protein of SARS-CoV-2 and PD of ACE2 were performed by molecular dynamics flexible fitting (MDFF) [14] of the published structure (PDB ID: 7C2L) and (PDB ID: 6M18) for majority. And the other parts were performed in the cryo-EM map with Phenix [15] and Coot [16] based on the focused-refined cryo-EM maps of all models with aromatic residues as landmarks, most of which were clearly visible in the cryo-EM map. Each residue was manually checked with the chemical properties taken into consideration during model building. Several segments, whose corresponding densities were invisible, were not modeled. Structural refinement was performed in Phenix with secondary structure and geometry restraints to prevent overfitting. To monitor the potential overfitting, the model was refined against one of the two independent half maps from the gold-standard 3D refinement approach. Then, the refined model was tested against the other map. Statistics associated with data collection, 3D reconstruction and model building were summarized in Supplemental information, Table S1.

For the model building of the conformation 1 of S-ACE2-B^0AT1 ternary complex, the atomic models of the S protein and the ACE2-B^0AT1 complex were docked into the local corresponding maps which had been fitted into the whole map, and then merged together to obtain the final docking model.

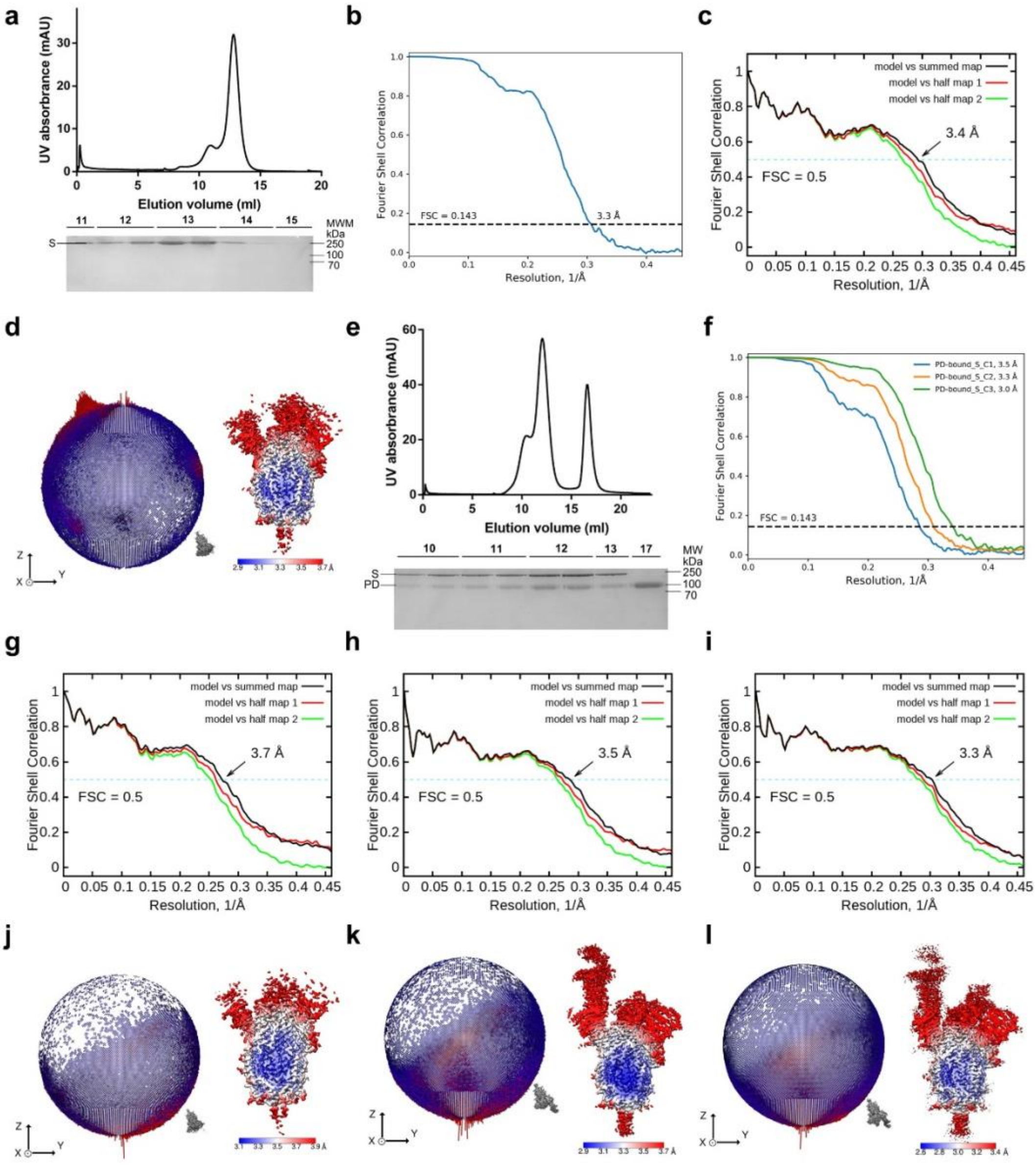

Supplementary information, Fig. S1| Cryo-EM analysis of native S and S-PD complex

a Representative SEC purification profile of S with GSAS mutations. **b** FSC curve of the overall structure of S protein. **c** FSC curve of the refined model of S protein versus the overall structure that it is refined against (black); of the model refined against the first half map versus the same map (red); and of the model refined against the first half map versus the second half map (green). The small difference between the red and green curves indicates that the refinement of the atomic coordinates is not enough overfitting.

d Euler angle distribution and local resolution map in the final 3D reconstruction of S protein. **e** Representative SEC purification profile of PD-bound S complex. **f** FSC curve of three conformation of PD-bound S complex, which the C1 shows as blue, C2 shows as orange and C3 shows as green. **g-i** FSC curve of the refined model of C1, C2 and C3 of PD-bound S complex in the order, which is the same as the **c**, respectively. **j-l** Euler angle distribution and local resolution maps for the 3D reconstruction of three conformation of PD-bound S complex in the order, respectively.

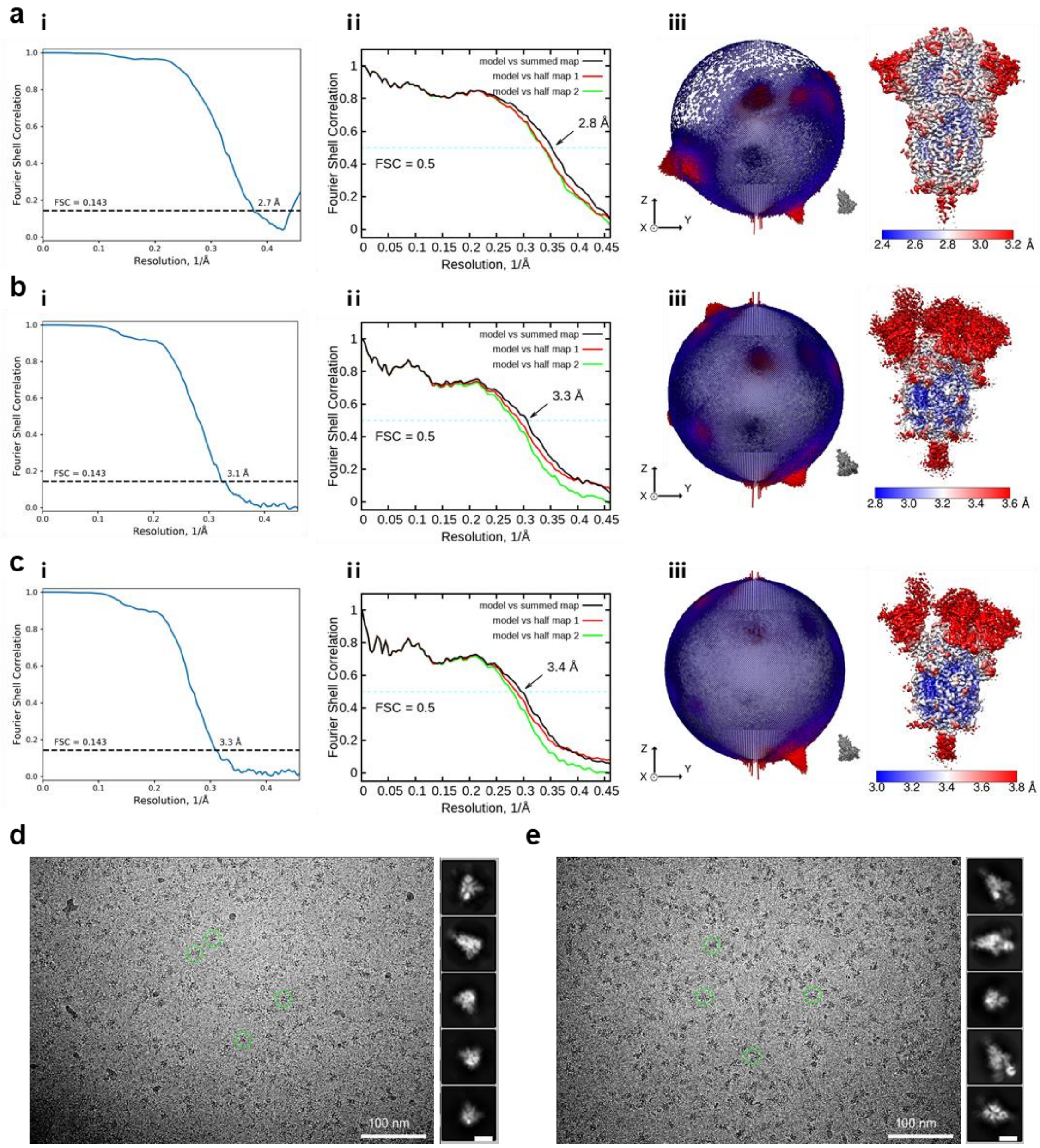

Supplementary information, Fig. S2| Cryo-EM analysis of S protein with different status

a Cryo-EM analysis of S protein in locked state. **b** Cryo-EM analysis of S(D614G). **c** Cryo-EM analysis of S(p, D614G). **i** FSC curve of the overall structure. **ii** FSC curve of the refined model versus the overall structure that it is refined against (black); of the model refined against the first half map versus the same map (red); and of the model refined against the first half map versus the second half map (green). The small difference between the red and green curves indicates that the refinement of the atomic

coordinates is not enough overfitting. **iii** Euler angle distribution and local resolution map in the final 3D reconstruction. **d** and **e** Representative cryo-EM micrograph and 2D class averages of cryo-EM particle images of S and PD-bound S(p) complex, respectively. The scale bar in 2D class averages is 10 nm.

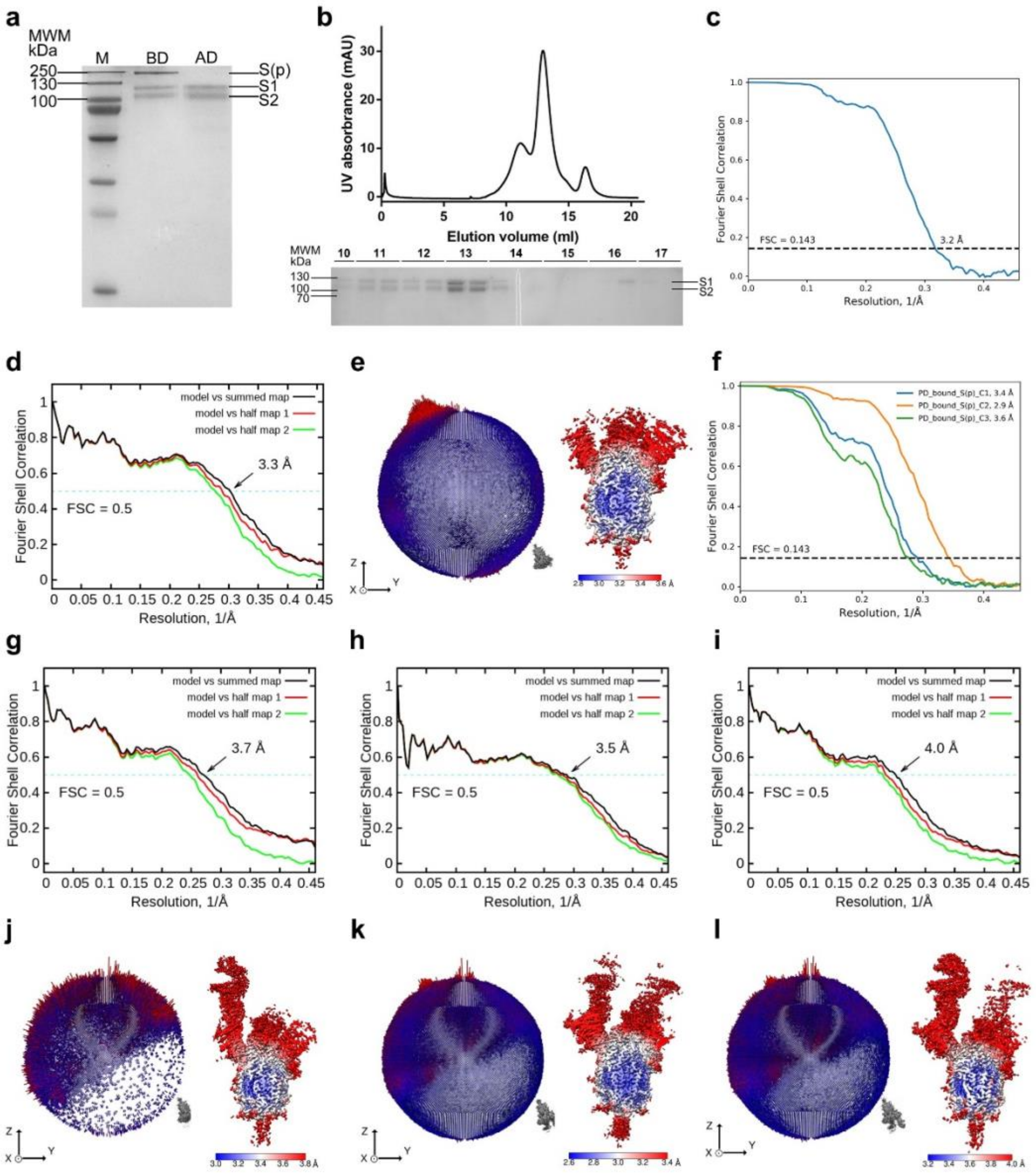

Supplementary information, Fig. S3| Cryo-EM analysis of S(p) protein and PD-bound S(p) complex

a SDS-PAGE of S(p) before and after trypsin digest. BD means before trypsin digest and AD means after trypsin digest. **b** Representative SEC purification profile of S(p) at trypsin-cleaved state. **c** FSC curve of the overall structure of S(p). **d** FSC curve of the refined model of S(p) versus the overall structure that it is refined against (black); of the model refined against the first half map versus the same map (red); and of the model refined against the first half map versus the second half map (green). The small

difference between the red and green curves indicates that the refinement of the atomic coordinates is not enough overfitting. **e** Euler angle distribution and local resolution map in the final 3D reconstruction of S(p). **e** Representative SEC purification profile of PD-bound S(p) complex. **f** FSC curve of three conformation of PD-bound S(p) complex, which the C1 shows as blue, C2 shows as orange and C3 shows as green. **g-i** FSC curve of the refined model of C1, C2 and C3 of PD-bound S(p) complex in the order, which is the same as the **c**, respectively. **j-l** Euler angle distribution and local resolution maps for the 3D reconstruction of three conformation of PD-bound S(p) complex in the order, respectively.

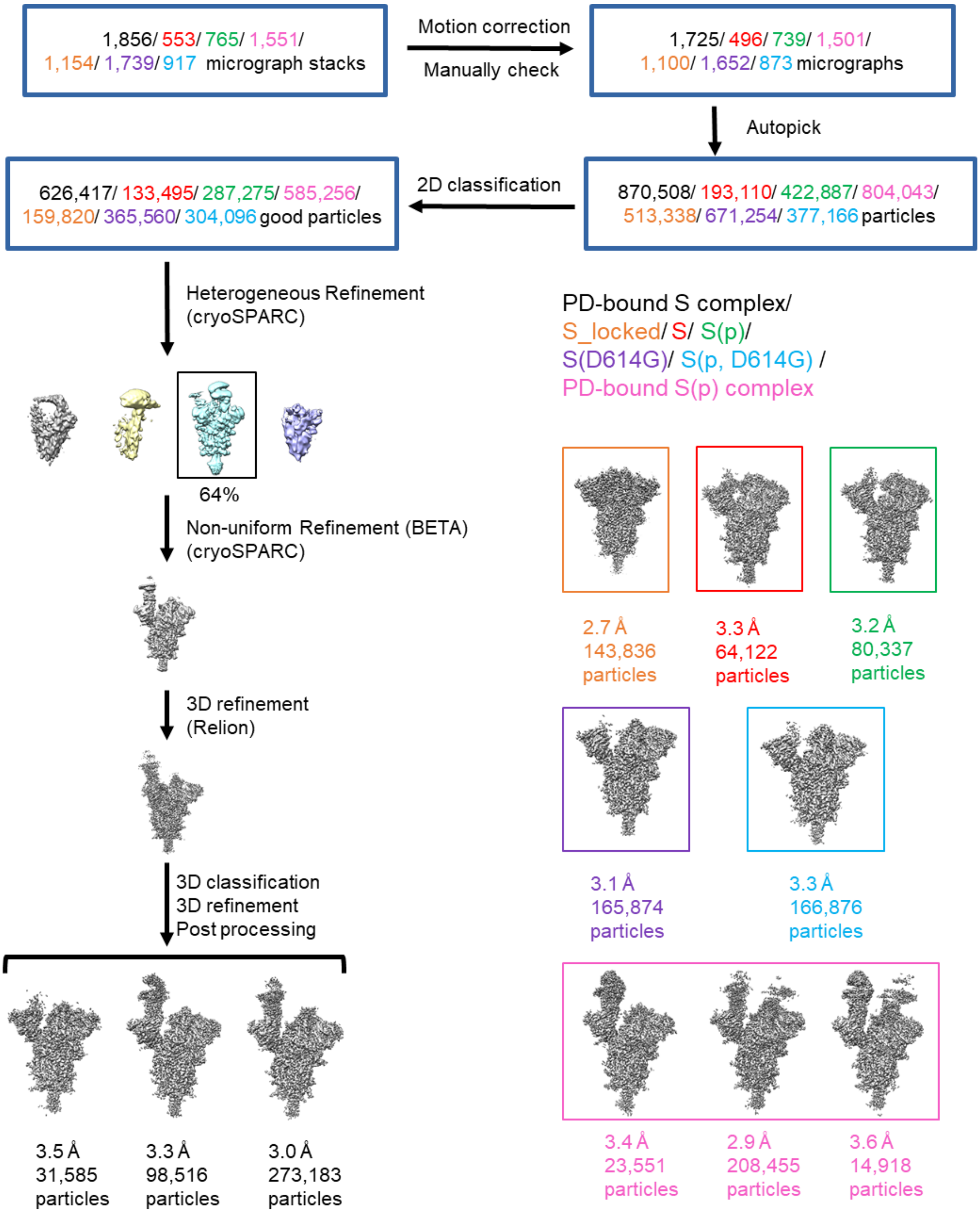

Supplementary information, Fig. S4| Flowchart for cryo-EM data processing.

Flowchart for cryo-EM data processing of all structure, please refer to the ‘Data Processing’ section in Methods for details.

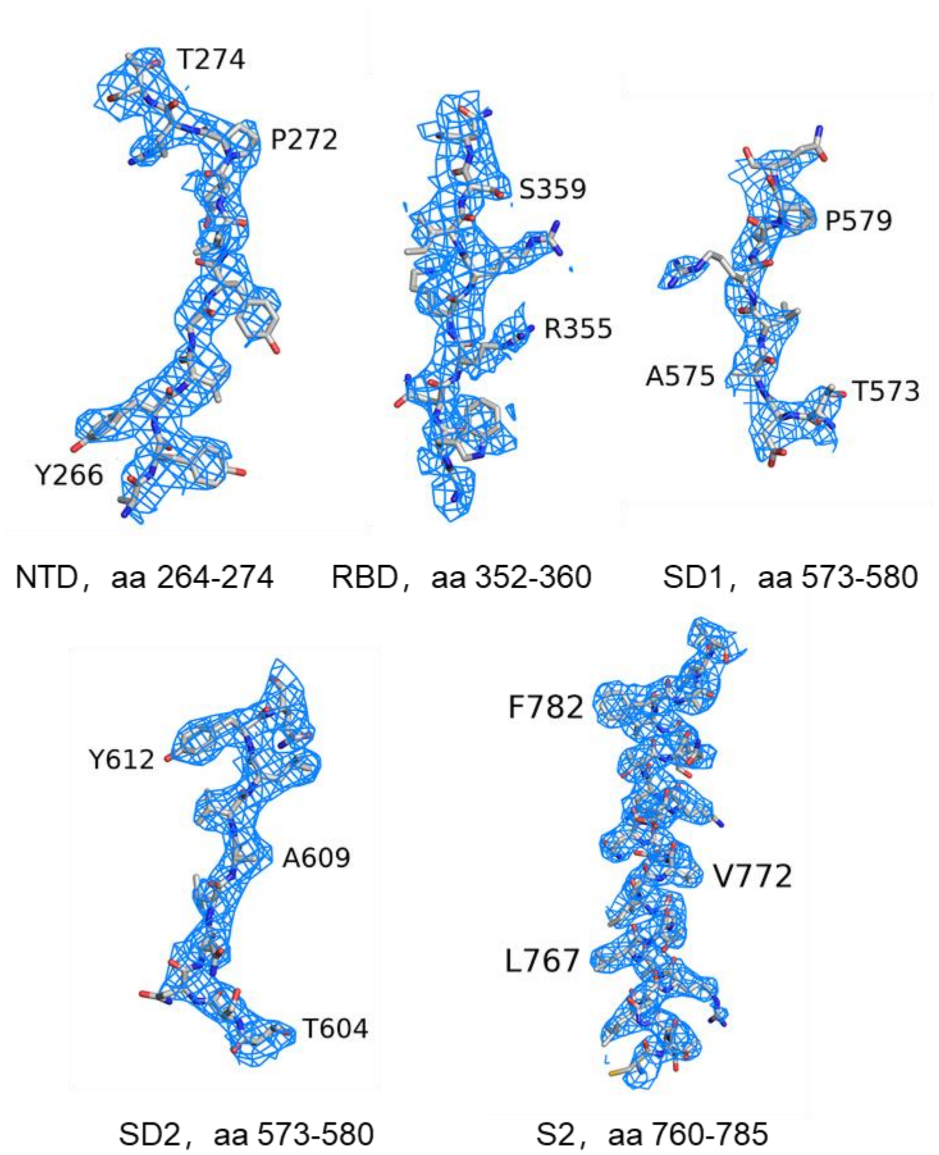

Supplementary information, Fig. S5| Representative Cryo-EM densities.

Shown here are the cryo-EM maps of indicated segments of PD-bound S complex C2. All densities are generated in PyMOL and contoured at 7 σ.

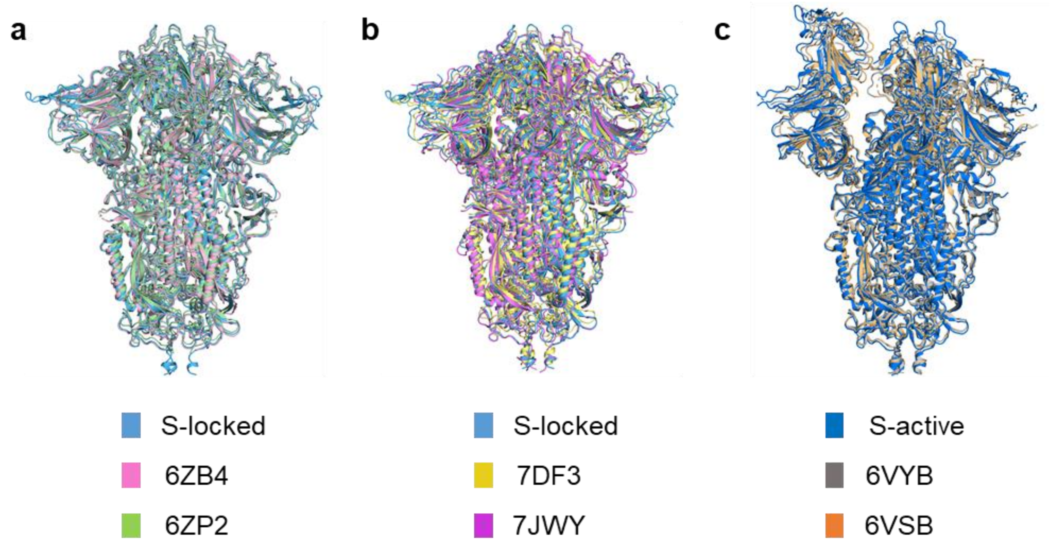

Supplementary information, Fig. S6| Structural comparison between S protein and reported structures

a and **b** Comparison between S-locked (cyan) and reported structures：6ZB4 (pink), 6ZP2 (green), 7DF3 (yellow) and 7JWY (magenta). **c** Comparison between S-active (blue) and reported structures: 6VYB (grey) and 6VSB (orange). There is no considerable structural difference between the compared items. S-locked means S protein in locked conformation and S-active means S protein in active conformation.

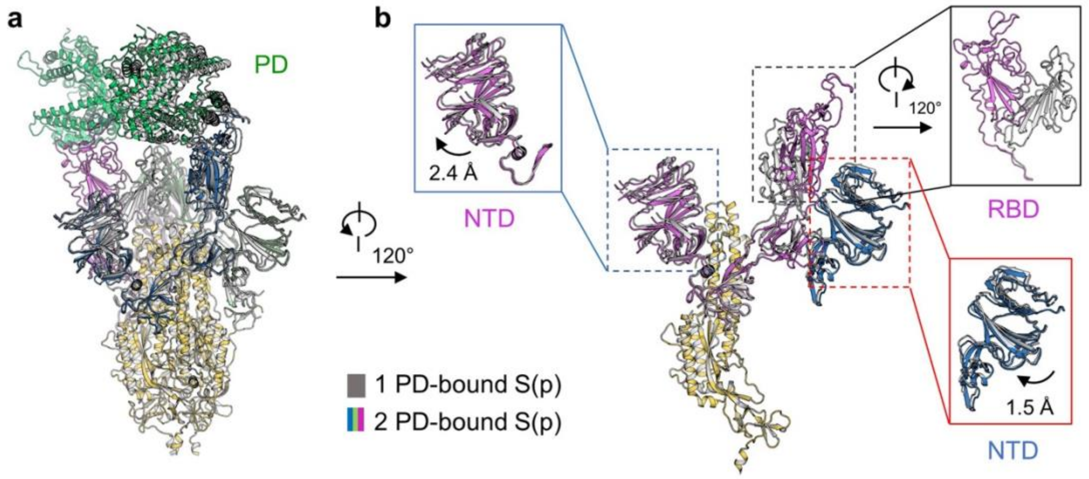

Supplementary information, Fig. S7| Comparison between 1 PD- and 2 PD-bound S(p).

a Overall alignment of the two structures. **b** The binding of the second PD to RBD of S(p) causes slight shift of NTD in the same protomer and in the anticlockwise protomer. The three protomers of the 2 PD-bound S(p) are shown in different colors (red, green, blue).

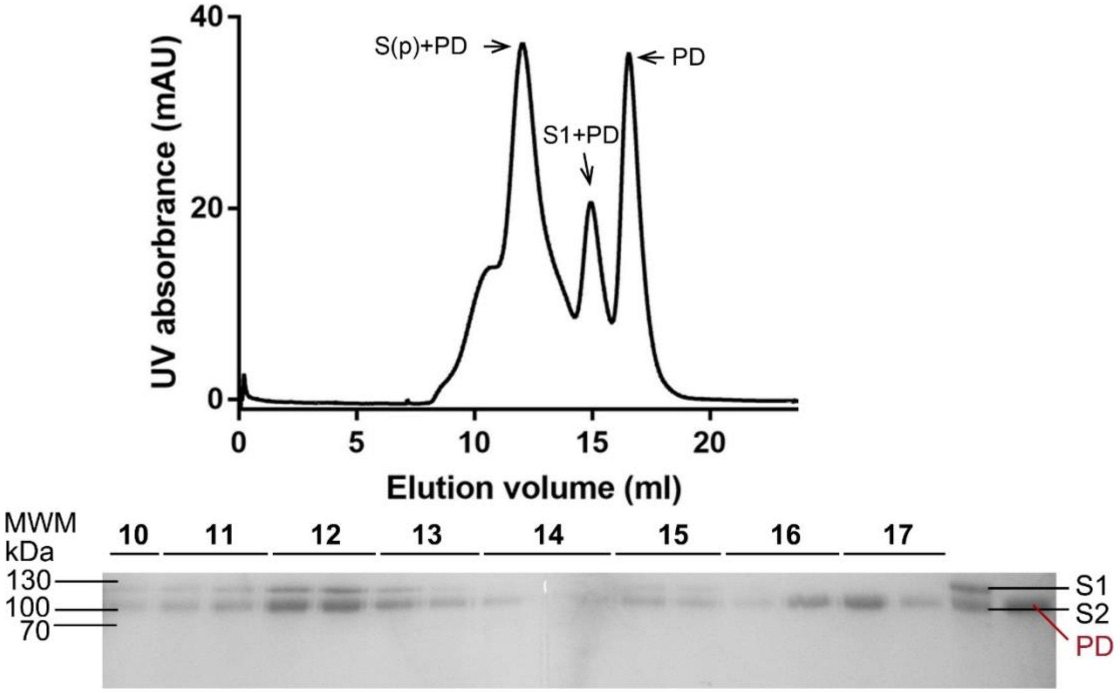

Supplementary information, Fig. S8| Representative SEC purification profile of S (p) after being incubated with PD.

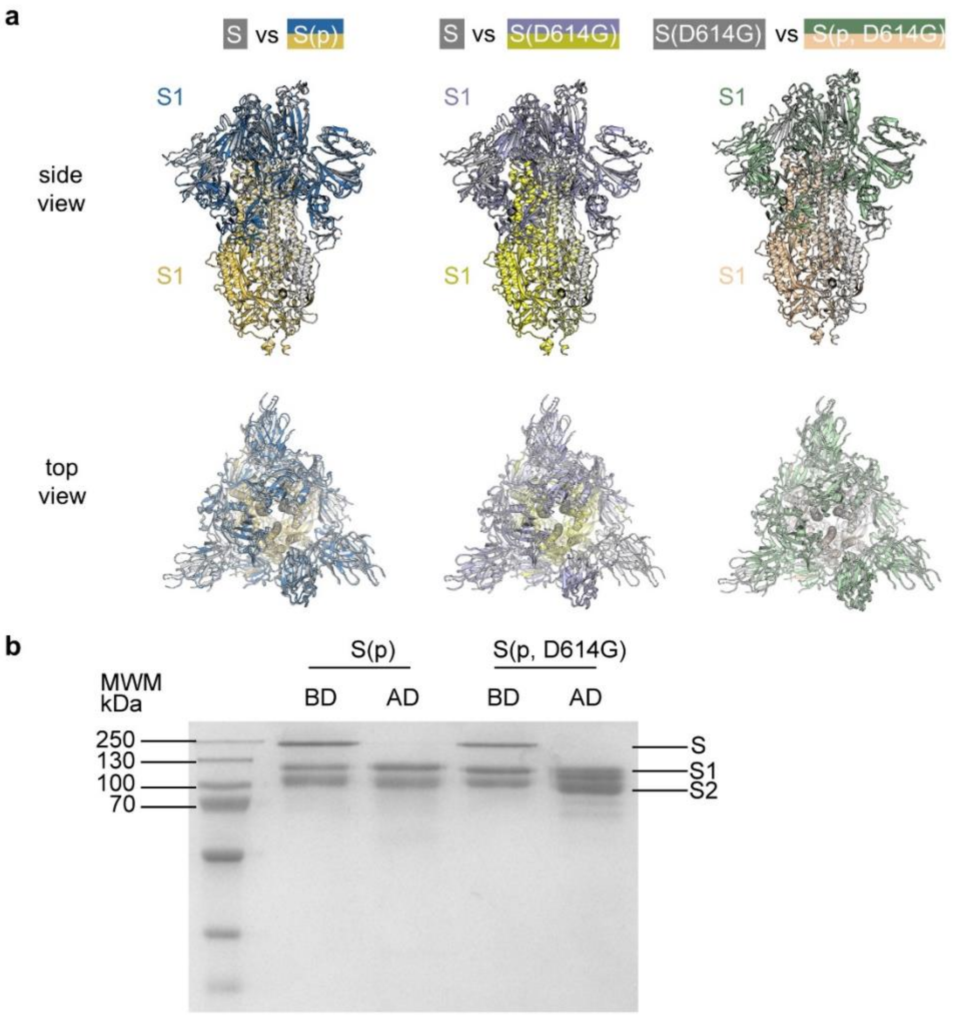

Supplementary information, Fig. S9| No significant difference between S, S(p), S(D614G) and S(p, D614G)

a Comparison between S and S(p) in left panel; comparison between S and S(D614G) in middle panel; comparison between S(D614G) and S(p, D614G) in right panel. **b** SDS-PAGE of S(p) and S(p, D614G) before and after trypsin-cleaving. BD means before trypsin-cleaving and AD means after trypsin-digest.

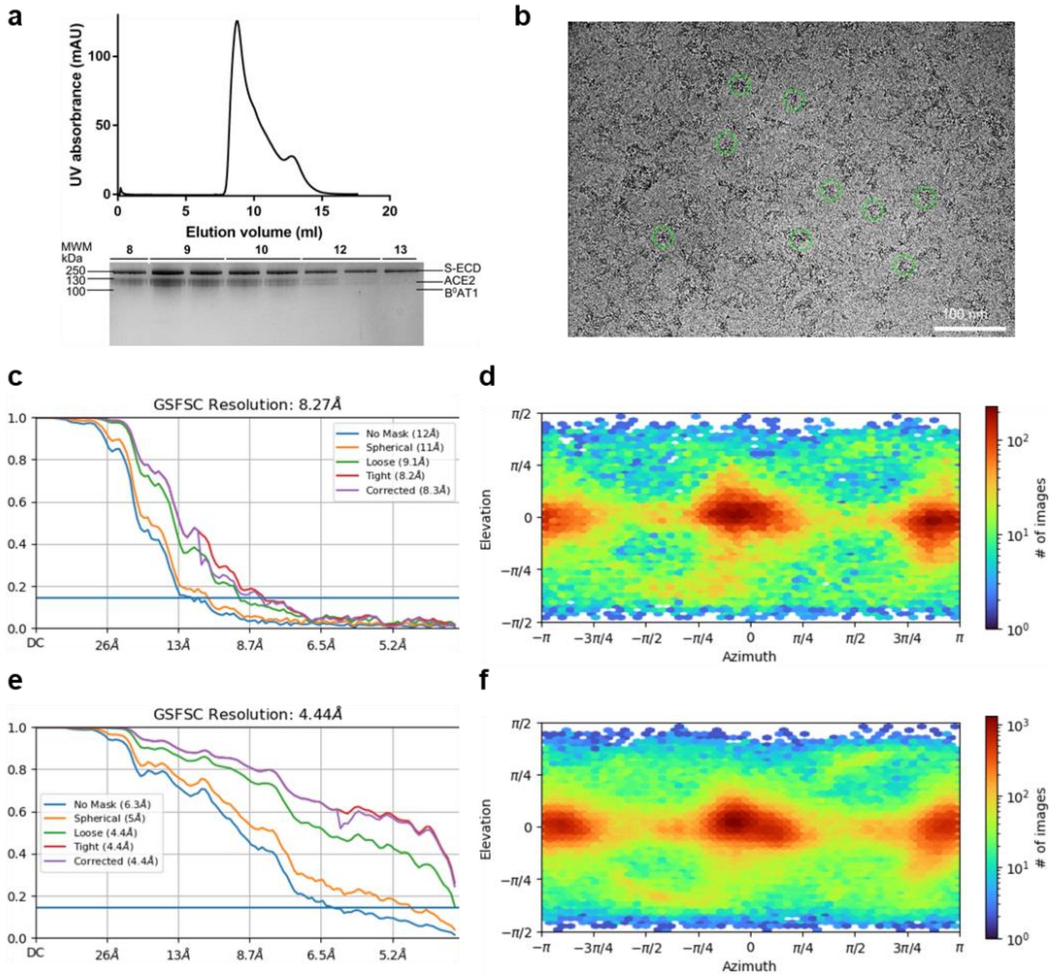

Supplementary information, Fig. S10| Cryo-EM images and flowchart for data processing of the S-ACE2-B^{0}AT1 ternary complex.

a Representative SEC purification profile of S-ACE2-B^{0}AT1 complex. **b** Representative cryo-EM micrograph of S-ACE2-B^{0}AT1 ternary complex. **c** and **e** FSC curve of conformation 1 and 2 of S-ACE2-B^{0}AT1 ternary complex, respectively. **d** and **f** Euler angle distribution in the final 3D reconstruction of conformation 1 and 2 of S-ACE2-B^{0}AT1 ternary complex, respectively.

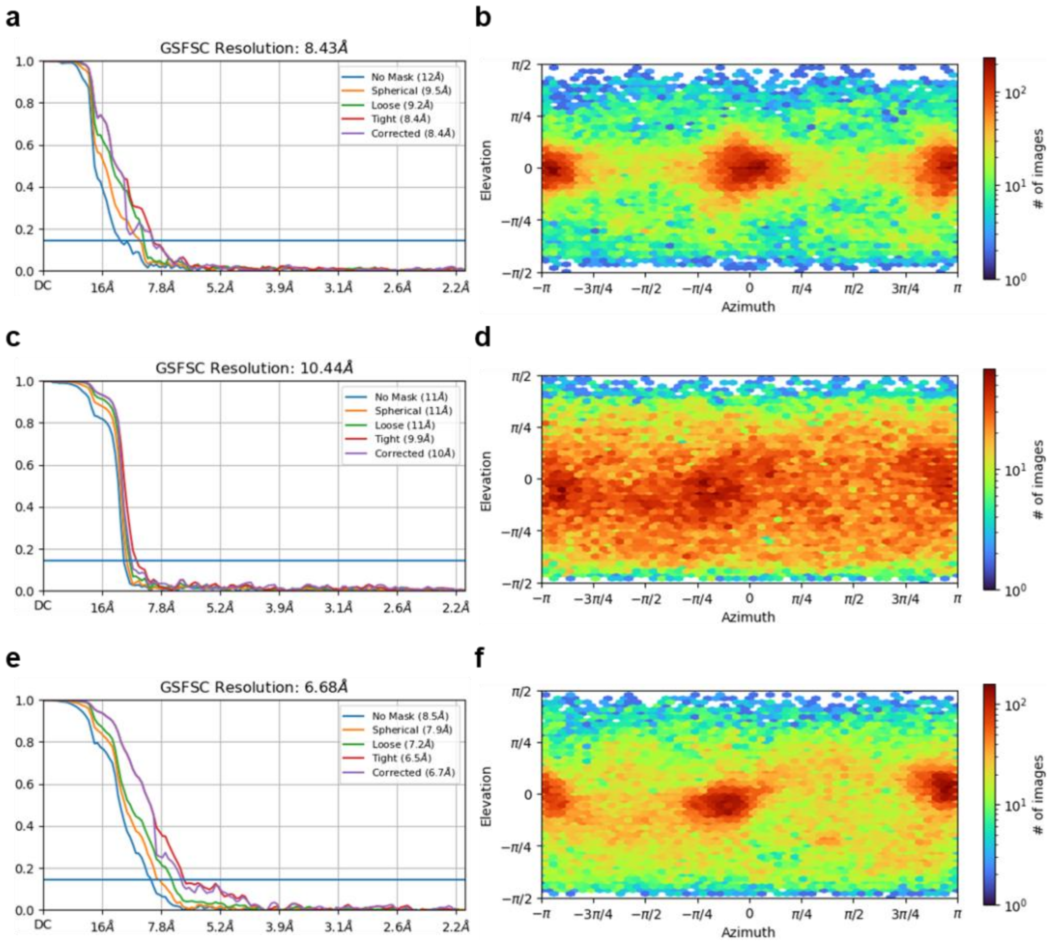

Supplementary information, Fig. S11| Cryo-EM images and flowchart for data processing of the S-ACE2-B^{0}AT1 ternary complex.

a, c and **e** FSC curve of conformation 1 of S-ACE2-B^{0}AT1 ternary complex focused on ACE2, the left S protein and the right S protein, respectively. **b, d** and **f** Euler angle distribution in the final 3D reconstruction of conformation 1 of S-ACE2-B^{0}AT1 ternary complex focused on ACE2, the left S protein and the right S protein, respectively.

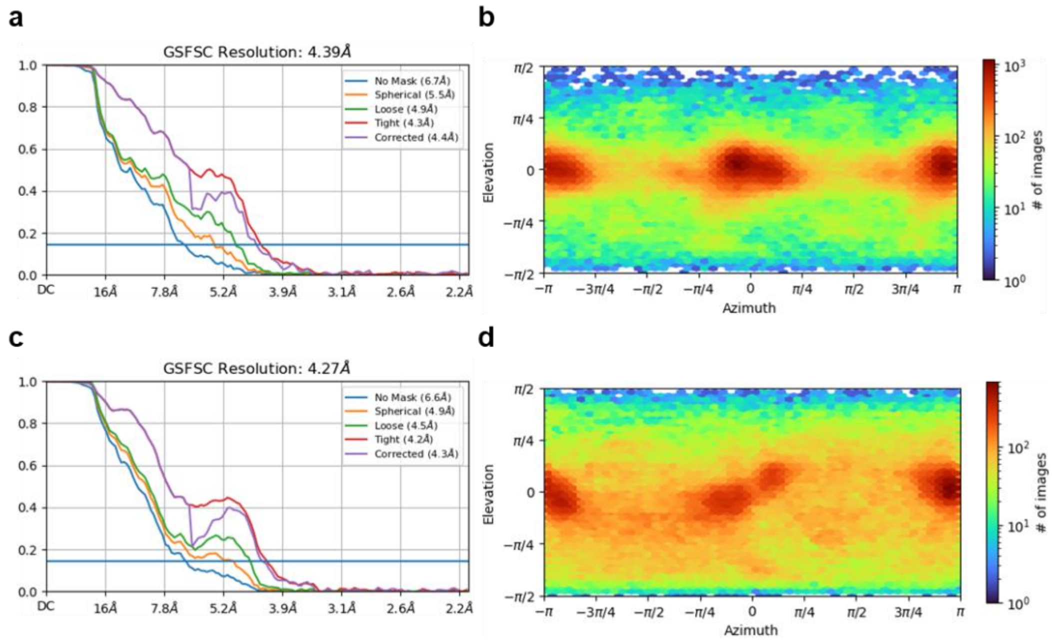

Supplementary information, Fig. S12| Cryo-EM images and flowchart for data processing of the S-ACE2-B^{0}AT1 ternary complex.

a and **c** FSC curve of conformation 2 of S-ACE2-B^{0}AT1 ternary complex focused on ACE2 and the right S protein, respectively. **b** and **d** Euler angle distribution in the final 3D reconstruction of conformation 2 of S-ACE2-B^{0}AT1 ternary complex focused on ACE2 and the right S protein, respectively.

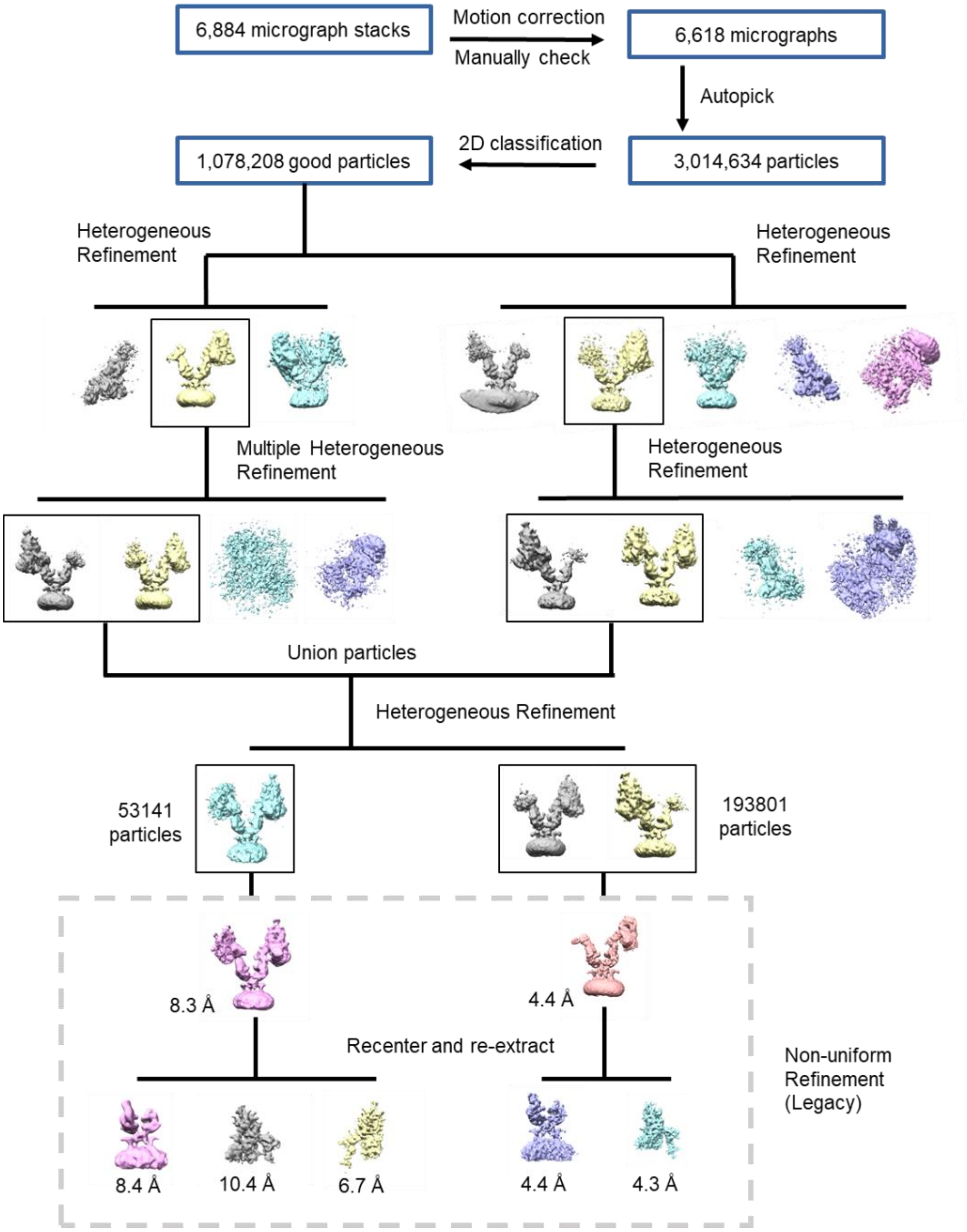

Supplementary information, Fig. S13| Flowchart for cryo-EM data processing of S-ACE2-B^{0}AT1 ternary complex.

Please refer to the 'Data Processing' section in Methods for details.

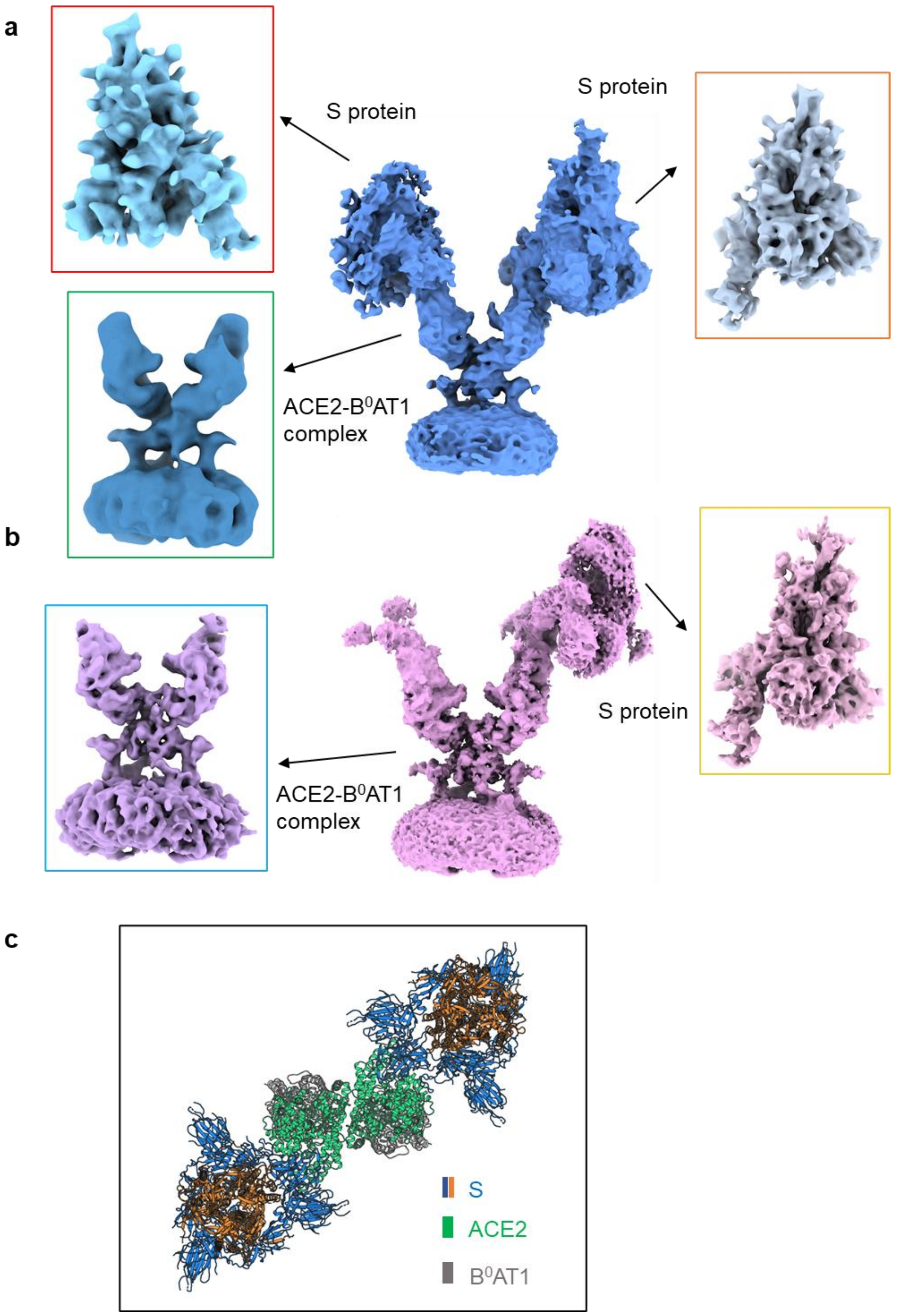

Supplementary information, Fig. S14| Reconstruction of S-ACE2-B0AT1 ternary complex.

a Conformation 1 of S-ACE2-B^{0}AT1 ternary complex. The surrounding maps represent focused refinement of ACE2, the left S protein and the right S protein. **b** Conformation

2 of S-ACE2-B^0AT1 ternary complex. The surrounding maps represent focused refinement of ACE2 and the right S protein. **c** Docking model of conformation 1 of S-ACE2-B^0AT1 ternary complex in top view.

Supplementary information, Video S1| Transition of the S protein from locked, closed (PBD 6VXX) to active conformation.

Supplementary information, Video S2| Comparison between the conformations of the S protein when PD is added.

Supplementary information, Video S3| Comparison between the conformations of the S(p) protein when PD is added.

Supplementary information, Table. S1| Cryo-EM data collection and refinement statistics.

**Data collection**					
EM equipment	Titan Krios (Thermo Fisher Scientific)				
Voltage (kV)	300				
Detector	Gatan K3 Summit				
Energy filter	Gatan GIF Quantum, 20 eV slit				
Pixel size (Å)	1.087				
Electron dose (e-/Å2)	50				
Defocus range (μm)	-1.2 ~ -2.2				
Number of collected micrographs	1,154	553	765	1,739	917
Number of selected micrographs	1,100	496	739	1,652	873
Sample	S_locked	S_active	S(p)	S(D614G)	S(p, D614G)
PDB code	7DWY	7DWZ	7DX0	7DX1	7DX2
EMDB code	EMD-30889	EMD-30890	EMD-30891	EMD-30892	EMD-30893
**3D Reconstruction**					
Software	cryoSPARC/ Relion				
Number of used particles	143,836	64,122	80,337	165,874	166,876
Resolution (Å)	2.7	3.3	3.2	3.1	3.3
Symmetry	C1				
Map sharpening B factor ($Å^2$)	-90				
**Refinement**					
Software	Phenix				
Cell dimensions (Å)	313.056				
Model composition					
Protein residues	3,297	2,913	2,913	2,913	2,913
Side chains assigned	3,297	2,913	2,913	2,913	2,913
Sugar	81	71	71	71	71
R.m.s deviations					
Bonds length (Å)	0.006	0.007	0.005	0.011	0.010
Bonds Angle (°)	0.795	0.952	0.845	1.049	0.997
Ramachandran plot statistics (%)					
Favored	96.14	92.28	93.26	90.40	91.49
Allowed	3.76	7.65	6.67	9.25	8.23
Outlier	0.09	0.07	0.07	0.35	0.28

Supplementary information, Table. S1, continued

**Data collection**						
EM equipment	Titan Krios (Thermo Fisher Scientific)					
Voltage (kV)	300					
Detector	Gatan K3 Summit					
Energy filter	Gatan GIF Quantum, 20 eV slit					
Pixel size (Å)	1.087					
Electron dose (e-/Å2)	50					
Defocus range (μm)	-1.2 ~ -2.2					
Number of collected micrographs	1,856			1,551		
Number of selected micrographs	1,725			1,501		
Sample	PD-bound S complex			PD-bound S(p) complex		
Conformation	C1*	C2*	C3*	C1*	C2*	C3*
PDB code	7DX3	7DX5	7DX6	7DX7	7DX8	7DX9
EMDB code	EMD-30894	EMD-30896	EMD-30897	EMD-30898	EMD-30899	EMD-30900
**3D Reconstruction**						
Software	cryoSPARC/ Relion					
Number of used particles	31,585	98,516	273,183	23,551	208,455	14,918
Resolution (Å)	3.5	3.3	3.0	3.4	2.9	3.6
Symmetry	C1					
Map sharpening B factor ($Å^2$)	-90					
**Refinement**						
Software	Phenix					
Cell dimensions (Å)	313.056					
Model composition						
Protein residues	2,913	3,544	3,544	3,544	4174	4209
Side chains assigned	2,913	3,544	3,544	3,544	4174	4209
Sugar	71	82	82	82	93	93
R.m.s deviations						
Bonds length (Å)	0.004	0.008	0.009	0.009	0.009	0.008
Bonds Angle (˚)	0.811	0.920	1.068	0.986	1.146	1.144
Ramachandran plot statistics (%)						
Favored	92.98	92.70	92.23	92.16	92.55	93.45
Allowed	6.95	7.21	7.71	7.76	7.43	6.47
Outlier	0.07	0.09	0.06	0.09	0.02	0.07

*, C1, C2 and C3 mean conformation 1, 2 and 3, respectively.

Supplementary References

1 Chi, X. *et al.* A neutralizing human antibody binds to the N-terminal domain of the Spike protein of SARS-CoV-2. *Science*, eabc6952, doi:10.1126/science.abc6952 (2020).

2 Yan, R. *et al.* Structural basis for the recognition of SARS-CoV-2 by full-length human ACE2. *Science* **367**, 1444, doi:10.1126/science.abb2762 (2020).

3 Lei, J. & Frank, J. Automated acquisition of cryo-electron micrographs for single particle reconstruction on an FEI Tecnai electron microscope. *Journal of structural biology* **150**, 69-80, doi:10.1016/j.jsb.2005.01.002 (2005).

4 Zheng, S. Q. *et al.* MotionCor2: anisotropic correction of beam-induced motion for improved cryo-electron microscopy. *Nature methods* **14**, 331-332, doi:10.1038/nmeth.4193 (2017).

5 Grant, T. & Grigorieff, N. Measuring the optimal exposure for single particle cryo-EM using a 2.6 A reconstruction of rotavirus VP6. *eLife* **4**, e06980, doi:10.7554/eLife.06980 (2015).

6 Zhang, K. Gctf: Real-time CTF determination and correction. *Journal of structural biology* **193**, 1-12, doi:10.1016/j.jsb.2015.11.003 (2016).

7 Zivanov, J. *et al.* New tools for automated high-resolution cryo-EM structure determination in RELION-3. *eLife* **7**, doi:10.7554/eLife.42166 (2018).

8 Kimanius, D., Forsberg, B. O., Scheres, S. H. & Lindahl, E. Accelerated cryo-EM structure determination with parallelisation using GPUs in RELION-2. *eLife* **5**, doi:10.7554/eLife.18722 (2016).

9 Scheres, S. H. RELION: implementation of a Bayesian approach to cryo-EM structure determination. *Journal of structural biology* **180**, 519-530, doi:10.1016/j.jsb.2012.09.006 (2012).

10 Scheres, S. H. A Bayesian view on cryo-EM structure determination. *Journal of molecular biology* **415**, 406-418, doi:10.1016/j.jmb.2011.11.010 (2012).

11 Punjani, A., Rubinstein, J. L., Fleet, D. J. & Brubaker, M. A. cryoSPARC: algorithms for rapid unsupervised cryo-EM structure determination. *Nature methods* **14**, 290-296, doi:10.1038/nmeth.4169 (2017).

12 Rosenthal, P. B. & Henderson, R. Optimal determination of particle orientation, absolute hand, and contrast loss in single-particle electron cryomicroscopy. *Journal of molecular biology* **333**, 721-745, doi:10.1016/j.jmb.2003.07.013 (2003).

13 Chen, S. *et al.* High-resolution noise substitution to measure overfitting and validate resolution in 3D structure determination by single particle electron cryomicroscopy. *Ultramicroscopy* **135**, 24-35, doi:10.1016/j.ultramic.2013.06.004 (2013).

14 Trabuco, L. G., Villa, E., Mitra, K., Frank, J. & Schulten, K. Flexible fitting of atomic structures into electron microscopy maps using molecular dynamics. *Structure (London, England : 1993)* **16**, 673-683, doi:10.1016/j.str.2008.03.005 (2008).

15 Adams, P. D. *et al.* PHENIX: a comprehensive Python-based system for macromolecular structure solution. *Acta crystallographica. Section D, Biological crystallography* **66**, 213-221, doi:10.1107/s0907444909052925 (2010).

16 Emsley, P., Lohkamp, B., Scott, W. G. & Cowtan, K. Features and development of Coot. *Acta crystallographica. Section D, Biological crystallography* **66**, 486-501, doi:10.1107/s0907444910007493 (2010).
